# Standardization of PD-L1 immunohistochemistry

**DOI:** 10.1038/s41379-021-00917-4

**Published:** 2021-09-10

**Authors:** Sandra Martinez-Morilla, Myrto Moutafi, David L. Rimm

**Affiliations:** Department of Pathology, Yale University School of Medicine, New Haven, CT USA

**Keywords:** Predictive markers, Immunotherapy

The immunohistochemistry (IHC) assays for PD-L1 have been a source of great uncertainty in both oncologists and pathologists. The wide range of FDA-approved assays with differential sensitivity and scoring system^1^, and the lack of success in harmonization of these assays^2,3^ has led to confusion in pathology labs. Currently, in breast cancer, the oncologist’s treatment plan must either be known in advance of the assay performance, or two non-concordant assays (Ventana SP142 and Agilent 22c3) must be performed since each qualifies patients for different PD-1 axis therapy (Atezolizumab and Pembrolizumab, respectively). Although the problem of different scoring systems is not easily resolved, in this issue of Modern Pathology, Sompuram et al. provide potential tools to standardize the sensitivity of the assays and to maintain lab-to-lab or week-to-week reproducibility of assessment of PD-L1 expression.

In this study, a new tool is described that allows evaluation of IHC assays using analytic parameters including limit of detection (LOD) and dynamic range of PD-L1. This National Institute of Standards (NIST)-based tool uses calibrators consisting of microbeads coated with a range of peptides representing the antigenic epitope portion of PD-L1 for each antibody/assay. In each spot of matrix containing beads on the slide, the number of molecules of the protein per bead has been previously determined spectroscopically. This system has been explored by the authors for a number of applications, including a recently published study with the calibrators for estrogen receptor earlier this year^4^, piloting methods that are then used for the PD-L1 calibrators toward standardization of many of the current PD-L1 assays. In the current study, the PD-L1 calibrators were used to compare four FDA-approved PD-L1 IHC assays and a series of LDT assays, among 41 laboratories from four different countries. After image analysis, they observed that the four FDA-approved PD-L1 assays could be grouped in three different levels of analytic sensitivity: SP263 - 22C3/28-8 - SP142 in order of decreasing analytic sensitivity (increasing LOD). In fact, this approach shows that in some labs the Ventana SP142 assay is analytically ten times less sensitive than the Ventana SP263 assay. The authors also show that the performance of the assessed LDT PD-L1 assays was similar to FDA-approved assays in many labs, with respect to LOD and analytic response curves.

Can this tool be used to harmonize the PD-L1 assay and potentially use a single assay to provide the correct information for each drug? Unfortunately, no. The reason is that the FDA approved PD-L1 assays have two components, the biochemical part of the assay which happens in the autostainer or on the slide, and the interpretive part, which happens when the pathologist converts what they see on the slide to a “score”. The score part cannot be addressed by this tool, but the biochemical part can!

**Fig. 1:**
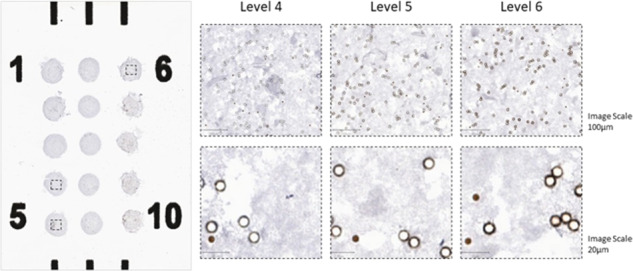
Representative image showing a panoramic view of the PD-L1 calibrator slide and three sequential levels of PD-L1 expression after DAB E1L3N LDT assay in two different magnification scales (100 µm top row and 20 µm bottom row). Level 4 corresponds to 150,157 PD-L1 molecules/bead, Level 5 287,131 molecules/bead and Level 6 642,523 molecules/bead. In this case Level 4 is below the LOD for this assay where Levels 5 and 6 are above. The small brown beads are used as a standard for automated reading. The larger beads show some refractility, but on the microscope, the last negative spot (Level 4) can be distinguished from the first positive spot (Level 5, the LOD). PD-L1 Programmed Death cell Ligand 1, DAB 3,3′-Diaminobenzidine, LDT Laboratory Development Test.

In spite of the common use of autostaining platforms, all of the PD-L1 IHC assays have shown to vary in intensity from week to week by as much as two SD^5^. This calibrator tool can be used to show week-to-week or month-to-month reproducibility of any assay and alert a lab director to a light or dark batch. Although Sompuram et al. do not show any images of the bead spots and only measure the beads using a stain intensity percentage (not explained in this paper but defined in Vani et al.^6^ as a complex MatLab algorithm), the inventor envisioned “by eye” assessment in clinical labs, where the beads appear increasingly brown by conventional light microscopic evaluation (see Fig. 1 produced in our lab). Using this tool, the IHC lab director could assess, by eye, the lightest positive spot, compared to the last negative spot and be sure that every stainer run in their lab results in the same threshold cut-point. This strategy should work regardless of which assay is used. Even though SP263 would show a different threshold than 22c3 and both would be more sensitive than SP142, the lab could be sure that they have reproducibility beyond that achievable with a single cell line control supplied by Agilent or the suggestion of a tonsil control in the label of the SP142 assay. Furthermore, using calibrators at the initial stages of assay development could lead to more accurate diagnostic tests that would stratify patients precisely during the clinical trial with standardization to NIST and actual numbers of molecules detected by the assay. The use of this tool could impact patient care in the near term as the patients are more likely to be correctly scored and receive the appropriate treatment based on an accurate and standardized measurement of the specific biomarker.

While the work of Sompuram et al. has the potential to change practice in pathology labs across the world, there are limitations. The most significant limitation is that this standardization approach does not address the problems associated with interpretation or scoring of PD-L1 IHC^7,8^. Until there are automated objective systems that can assess PD-L1 expression levels and localization, that issue is likely to generate a lot of publications but will not be easily solved. Another limitation is the substrate itself. While the peptides are designed to carefully mimic the antibody recognition sequence, the antigen is synthetic and may not exactly mimic the antigen seen in real human tumor tissue. However, this limitation should not affect the key utility of this tool as a standard. Finally, the ability to easily define the PD-L1 expression threshold bead number can be affected by the counterstain. Good laboratory practice, with minimal hematoxylin counterstain can address this limitation.

In summary, Sompuram et al. have produced a NIST-standardized calibrator tool that can solve the biochemical aspects of the variability of the PD-L1 assays, and its incorporation into IHC labs is likely to increase the chances that the right patients get the right drugs.
